# Pharmacokinetics of a peroral single dose of two long-acting formulations and an aqueous formulation of doxycycline hyclate in horses

**DOI:** 10.1186/1751-0147-55-21

**Published:** 2013-03-08

**Authors:** Heidi Zozaya, Lilia Gutierrez, Maria Josefa Bernad, Hector Sumano

**Affiliations:** 1 Department of Physiology and Pharmacology, School of Veterinary Medicine, National Autonomous University of Mexico (UNAM), Av. Universidad 3000, Coyoacan Mexico City 04360, Mexico; 2 Department of Pharmacy, School of Chemistry, National Autonomous University of Mexico (UNAM), Mexico City, Mexico

**Keywords:** Doxycycline, Long-acting, Horses, Pharmacokinetics, Oral-administration

## Abstract

**Background:**

Doxycyline (Dox) is a semisynthetic antibacterial drug with pharmacological advantages over its parent drug (tetracycline) in the treatment of various bacterial diseases in horses. Yet, at present a horse-customized pharmaceutical formulation is not available. Based on its pharmacokinetic/pharmacodynamic (PK/PD) ratio, Dox is considered a time-dependent antibacterial drug and ideally expected to achieve sustained plasma drug concentrations both at or slightly above the minimal inhibitory concentration (MIC) level for as long as possible between dosing intervals. Hence, the objective of this study was to formulate two long-acting (LA) doxycyline hyclate (Dox-h) formulations for oral administration and define their pharmacokinetics in non-fasted adult horses to obtain better bioavailability and longer mean residence time, features needed to comply better with its pharmacokinetic/pharmacodynamic (PK/PD) ratios.

**Results:**

Pharmacokinetic parameters were determined after the oral administration of a single 10 mg/kg bolus dose of two 20% Dox-h formulations: one based on a β cyclodextrin (Dox-β) matrix and a second one on a poloxamer (Dox-pol) matrix. The results were compared with the pharmacokinetics of a single 10 mg/kg bolus oral dose of a freshly made aqueous Dox-h solution (Dox-a). Dox-pol showed the greatest values for relative bioavailability (548%); maximum serum concentration (Cmax) value was 1.3 ± 0.7 μg/mL with time to reach the Cmax (Tmax) of 5.9 ± 1.7 h, area under the curve (AUC) of 17.0 ± 2.2 μg h/ml and elimination half-life (T½ β) of 4.9 ± 1.0 h.

**Conclusions:**

Considering a minimal inhibitory concentration MIC of 0.25 μg/mL, clinically effective plasma concentrations might be obtained for up to 24 h administering Dox-pol. This is an oral paste formulation that might optimize the use of Dox-h in horses in terms of PK/PD ratio congruency, and it is likely that it may also improve prescription compliance due to its ease of administration.

## Background

Doxycycline hyclate (Dox-h), a semi-synthetic analog of tetracycline, offers several pharmacological advantages over the parent drug (tetracycline) in horses, mainly higher oral bioavailability, higher tissue penetration, a larger volume of distribution and exhibits a more potent antimicrobial activity [1-5]. Additionally, Dox-h is better tolerated than other tetracyclines in this species; hence, the risk of enterocolitis and diarrhea is milder and/or infrequent [6,7].

**Figure 1:**
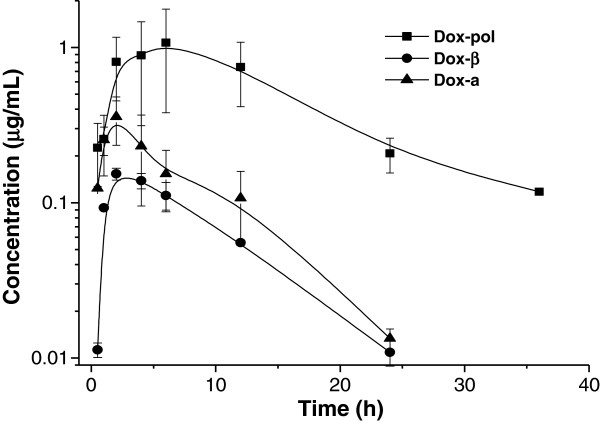
Concentration vs time profile of doxycycline (Dox) in serum after administration of three experimental oral formulations in horses.

The intramuscular and subcutaneous administration of Dox-h can cause extreme local pain, irritation and tissue necrosis and these routes are therefore not recommended [7,8]. The intravenous use should also be avoided, as it can cause supraventricular tachycardia, systemic arterial hypertension, clinical signs of discomfort, cardiovascular collapse and even death in horses [9,10].

The pharmacokinetics of an aqueous solution of Dox-h administered orally (PO) has been determined in adult horses [1,6,11,12] and in foals [2]. In these studies, Dox-h was administered either dissolved in water via nasogastric tube or as a top dressing at doses ranging from 3 mg/kg to 20 mg/kg every 12 (q12h) or every 24 hours (q24h). Davis et al. [6] determined that after administering single or multiple doses of Dox-h (20 mg/kg) via nasogastric tube, the time to maximum concentration (Tmax) was 1.6 ± 1.3 x`h, the maximum concentration (Cmax) was 1.7 ± 0.3 μg/mL and elimination half-life (T½β) was 12.07 ± 3.1 h. Plasma protein binding was 81.7 ± 2.4%. These authors concluded that Dox-h administered at a dosage of 20 mg/kg PO q24h will result in drug concentrations adequate for inhibiting intracellular bacteria and bacteria with minimal inhibitory concentration (MIC) equal or higher than 0.25 μg/mL. On the other hand, Bryant et al. [1] concluded that Dox-h at a dose of 10 mg/kg PO q12 h could be appropriate for treating infections caused by susceptible (MIC < 0.25 μg/ml) gram positive microorganisms. Yet, Davis et al. [6] and Womble et al. [2] consider that the therapeutic value of oral Dox-h in adult horses is limited due to its low bioavailability. Winther et al. [12] reported an estimated oral bioavailability of Dox-h of 17% after intragastric administration and 6% after topdressing administration in non-fasted adult horses. Similarly, these authors consider that if the oral bioavailability of Dox-h could be enhanced, this antimicrobial drug might be a valuable resource for the treatment of lower airway infections in horses. Additionally, a high local drug concentration of Dox-h in the stomach causes gastric irritation and nausea in humans [13,14] and it has been shown that retarding the release of Dox-h diminishes the incidence of gastrointestinal adverse side effects [15].

Considering the above, the objective of the present study was to formulate and define the pharmacokinetics of two long-acting (LA) Dox-h formulations intended for oral administration in horses, with the aim of improving its bioavailability and its gastrointestinal tolerance in an attempt to enhance the value of Dox-h as an antimicrobial drug in equine medicine.

## Methods

The study was conducted in Mexico City campus of the School of Veterinary Medicine at the National Autonomous University of Mexico (UNAM). The study was approved by the Postgraduate Committee of Research, Care, and Use of Experimental Animals in accordance with its regulations [16].

Ten healthy adult Quarter Horses (three mares and seven geldings) weighing a mean of 450 ± 22.4 kg were included in the study. The animals were considered clinically healthy on the basis of physical examination and standard hematological and biochemical tests. The horses had not been medicated with any antimicrobial agent for at least 30 days before enrollment in the study. They were maintained on a diet of oat hay and feed concentrate and had *ad libitum* access to water throughout the study.

Three preparations were assessed; two experimental LA formulations and an aqueous one. The first LA formulation was the Dox-h in a poloxamer base (Dox-pol; 200 mg/mL), prepared as follows: first Dox-h powder (PARFARM S.A., Mexico) was made soluble in distilled water. Then, a reverse gel copolymer polyoxypropile–polyoxyethylene poloxamer 407 (Lutrol micro 127 MP (BASF Germany) was added and stirred vigorously and constantly at 4°C. The mixture was protected from sunlight and maintained at 2 – 4°C during 24 h. It was then further homogenized to obtain a clear solution. Carbomer 934P (Carbopol 934P, Lubrizol USA) was added to increase viscosity. After stirring continuously during 30 minutes, it was considered ready when a microemulsion was formed. This point could be determined when the mixture clarified. Xanthan gum (Padoquimia S.A., Mexico) was then added to obtain a suspension with a paste-like consistency ready to use. Finally, 35 mL syringes were filled up with the 20% Dox-pol formulation, protected from light and the formulation used within three days after preparation.

The Dox-h–β cyclodextrin (Dox-β) on a poloxamer base formulation was prepared, by first forming complexes of Dox-h 20% (w/v) with β-cyclodextrin (Cerestar Pharmaceutical Excipients, U.S.A.). For this purpose the kneading method was used [17]. The ingredients were first mixed in a mortar to obtain a homogeneous paste. Then, Dox was added slowly. The mixture was further grounded for 30 min and an appropriate quantity of water was added to maintain a paste-like consistency. It was then dried in an oven at 40-50°C for 24 h. The dried complex was pulverized into a fine powder, which was then diluted in water. This mixture was then included in a reverse gel copolymer polyoxypropile–polyoxyethylene poloxamer 407 (Lutrol micro 127 MP (BASF Germany) under constant stirring at 4°C. The preparation was regarded as ready when a micro-emulsion is formed and this could be pin-pointed when the mixture clarified. Xanthan gum (Padoquimia S.A., Mexico) was then added to obtain a suspension with a paste-like consistency ready to use. Finally, 35 mL syringes were filled up with the 20% Dox-β formulation, protected from light and the formulation used within three days after preparation. This experiment does not intend to present stability studies for this preparation, and has no proprietary restrictions.

Finally, an aqueous formulation of Dox-h (Dox-a) was prepared from powdered Dox-h by diluting it in sterile distilled water, obtaining a 20% final solution and immediately administered to the horses.

Individual dose vs. pharmaceutical preparation compliance was calculated to have 5.5, 4.2 and 2.8% error from the set dose of 10 mg/kg in the Dox-β, Dox-pol and Dox-a (1 mL/20 kg of body weight in all cases), as assessed by determining Dox-h concentration in all three preparations, taking 4 random test samples of each group. Determination of Dox-h in by HPLC with UV detection in these pharmaceutical samples was carried out as described by Axisa et al. [18].

A longitudinal crossover (3 x 3 x 4) study design was employed with washout periods of 21 days. Each horse was individually weighed and dosed with the Dox-a preparation via nasogastric tube at a dose of 10 mg/kg in a volume of approximately 30 mL. After the washout period, each horse of the same experimental group was moved to the next group and dosed at 10 mg/kg with either Dox-β (30 mL approximately), or Dox-pol, also at 10 mg/kg in a volume of 30 mL approximately. These latter groups received their dose as a paste, placed in the interdental space aided by a long tipped syringe. In all three groups adverse gastrointestinal drug reactions i.e., colic, diarrhea and other signs of abdominal discomfort were sought for hourly during the day.

Determination of pharmacokinetic values was accomplished through serial blood sampling.

To achieve accurate intervals between administration of the drug and collection of serum, a 16-gauge, permanently heparinized catheter was inserted into a jugular vein and glue-fixed on each horse. Blood samples were obtained before administration of any of the formulations (time 0), and after the administration of each of the preparations, at 0.25, 0.5, 1, 2, 4, 6, 8, 10, 12, 24 and 48 h. Blood samples were obtained by removing 5 mL of heparin-containing blood from the catheter, discarding it, and then collecting additional 5 mL of blood, which were placed into 10 mL test tubes with no anticoagulant. Blood was allowed to clot at room temperature (20°C) during 30 minutes and then centrifuged at 1500 RPM for 15 minutes. Serum was harvested, frozen in liquid nitrogen and stored not more than 7 days until analyzed.

Serum Dox concentrations were determined both by high performance liquid chromatography (HPLC) as described by Axisa et al. [18] and through the modified agar diffusion analysis, described by Abd El-Aty et al. [19]. For the former analytical analysis, the intra-assay coefficient of variance was < 1.9 and interassay error was < 1.8. The analytic assay was linear over a range of concentrations from 0.1 to 10 μg/mL. Mean ± 1 SD recovery was 94 ± 2% (*r* = 0.97). Limit of detection was 0.07 μg/mL, and limit of quantification was 0.1 μg/mL.

For the modified agar diffusion analysis, *Bacillus cereus* (ATCC-11778) was used as a test organism grown on Mueller-Hinton agar (MCD LAB, S.A. de C.V., Mexico City). The intra-assay coefficient of variance was < 4.8 and inter-assay error < 4.6. The analytical assay was linear over a range of concentrations from 0.04 to 10 μg/mL, with a percent recovery of 93 ± 2 and a correlation coefficient (r) of 0.97 ± 0.1. Limit of detection was 0.005 μg/mL and limit of quantification was 0.01 μg/mL. Compliance between both methods to determine serum concentrations of Dox-h was carried out using doxycycline-spiked horse serum samples and processed by the two analytical techniques. Subtraction of recover percentages revealed an error of no more than 6.2%.

A computerized curve stripping program (PK Analyst for Windows, MicroMath, St. Louis, MO) was used to fit and analyze the concentration-versus-time profiles for each horse and the mean values for each group. Models of best fit (*r* ≥ 0.99) were chosen after analysis by use of residual sum of squares and the minimal Akaike’s information criterion. The best fit for Dox-a was obtained by using a 2 compartment model with first-order input and first order output in accordance with the following equation:

$$\begin{array}{l} \text{Concentration}\;\left(\text{Time}\right)=Ae^{-}{}^{a\;\text{Time}}+Be^{-}{}^{\beta \;\text{Time}}\\ \;+\;Ce^{-\text{KAB}\;\text{Time}} \end{array}$$

Pharmacokinetic variables obtained for Dox- a were: elimination rate constant (Kel), absorption rate constant (Kab), area under the curve (AUC), half life of the elimination phase (T ½ β), absorption half life (T½ ab), mean residence time (MRT), mean residence time to infinity (MRT _0_ - _∞_), area under the curve to infinity (AUC_0_ - _∞_) and area under the moment curve to infinity (AUMC_0_ - _∞_).

The best fit for Dox-pol and Dox-β was obtained by using a one-compartment model with first-order input and first order output in accordance with the following equation:

$$\text{Concentration}\;\left(\text{Time}\right)={\frac{\text{Dose}\;K_{\mathrm{AB}}}{\text{Volume}\;K_{\mathrm{AB}}-K_{\text{elim}}}}^{e^{K_{\mathrm{el}}\;\text{Time}}}{}^{–e^{K_{\mathrm{AB}}\;}{}^{\text{Time}}}$$

The pharmacokinetic values obtained for Dox-pol and Dox-β were: elimination rate constant (Kel), absorption rate constant (KAB), area under the curve (AUC), area under the first moment of the concentration-time curve (AUMC), half life of the elimination phase (T½β), absorption half life (T½ab), time when the concentration reaches its maximum (Tmax), the maximum concentration (Cmax), mean residence time (MRT), mean residence time to infinity (MRT _0 - ∞_), area under the curve to the last time point (AUC _0 - ∞_ ) and area under the moment curve to infinity (AUMC_0 - ∞_ ). Table 1 summarizes the pharmacokinetic variables obtained. Data showed no normal distribution for all three groups and are presented as mean ± standard deviation of 10 observations for each parameter. Statistical comparison was made by Kruskal-Wallis and Dunn test.

**Table 1:** Pharmacokinetic variables for doxycycline hyclate (Dox-h) in horses after the oral administration of three experimental formulations

	**Dox-a**	**Dox-pol|**	**Dox-β**
	**Mean ± SD**	**Mean ± SD**	**Mean ± SD**
AUC (μg · h/mL)	3.1 **±** 0.2 ^a^	17.0 ± 2.2^b^	1.5 ± 0.1 ^c^
AUMC (μg · h^2^/mL)	35.2 **±** 1.2 ^a^	208.3 ± 31.1^b^	12.3 ± 0.1 ^c^
AUC_0_**-**_∞ _(μg · h/mL)	3.0 ± 0.2 ^a^	16.1 ± 4.8 ^b^	1.5 ± 0.9 ^c^
AUMC_0 _**-**_∞ _(μg · h^2^/mL)	31.2 ± 2.3 ^a^	171.3 ± 5.8 ^b^	12.3 ± 0.5 ^c^
MRT (h)	11.3 ± 4.4 ^a^	12.2 ± 4.2^a^	8.1 ± 2.1 ^a^
MRT_0 _**-**_∞ _(h)	10.2 ± 2.6 ^a^	10.7 ± 2.1 ^a^	8.1 ± 1.2 ^a^
T½β (h)	2.8 ± 0.9^a^	4.9 ± 1.0^b^	4.2 ± 0.9^b^
T½_ab_(h)	1.2 ± 0.2 ^a^	3.5 ±1.2 ^b^	1.4 ± 0.1 ^a^
K_el_ (h^-1^)	0.2 ± 0.0 ^a^	0.1 ± 0.1 ^a^	0.2 ± 0.1 ^a^
Kab (h^-1^)	1.6 ± 0.2 ^a^	0.2 ± 0.2 ^b^	0.5 ± 0.3 ^c^
α (h^-1^)	1.2 ± 0.0	-	-
β (h^-1^)	9.2 ± 0.0	-	-
Cmax (μg/mL)	0.3 ± 0.1 ^a^	1.3 ± 0.7 ^b^	0.2 ± 0.0 ^c^
Tmax (h)	2.2 ± 0.4 ^a^	5.9 ± 1.7 ^b^	3.4 ± 0.6 ^c^
Frel (%)^*^	-	548% ^a^	48% ^b^

^a,b,c, ^The values within a row with no common superscript differ significantly (P < 0.05).

^* ^Mean of means.

*AUC*, Area under the concentration-time curve; *AUC*_*0*_***-***_*∞*_, Area under the concentration –time to infinity; *AUMC*, Area under the first moment of the concentration-time curve; *AUMC*_*0*_***-***_*∞*_, Area under the first moment of the concentration-time curve to infinity; *MRT*, Mean residence time; *MRT*_*0*_***-***_*∞*_, Mean residence time to infinity; *α*, distribution hybrid rate constant; *β*, elimination hybrid rate constant; *A, B,*, zero time intercepts of the distribution and post-distribution phases; *K*_*el*_, Elimination rate constant from the central compartment; *Kab*, Absorption rate constant; *T*_*½ab*_, Absorption half-life; *T*_*½β*_, Elimination half-life; *Cmax*, Calculated maximum plasma concentration; *Tmax*, Time of maximum plasma concentration; *Frel*, Relative bioavailability.

Relative bioavailability was calculated comparing the long acting formulations with the Dox- a preparation, by using the following equation as described by Sabnis [20]:

$$\begin{array}{l} \text{Relative}\;\text{bioavailability}\;\left(\text{Frel}\right)=\left(\text{AUCDox}-\text{pol}/\text{AUCDox}-a\right)\\ \;\times \;100 \end{array}$$

The degree of plasma protein binding of Dox was carried out in vitro as described by Singhvi et al. [21]. Dox-enriched plasma samples were spiked with 0.1, 0.5, 1, 5, 10, and 20 μg/mL of Dox and 1 mL was added to commercial ultrafiltration Waters Oasis solid-phase extraction cartridges (Waters Associates, Milford, MA). The ultrafiltrate was centrifuged at 1,200 × *g* for 30 min at 37°C to further separate plasma proteins. This resulted in an ultrafiltrate volume of at least 200 μL that was frozen until assayed. The resulting filtrates were used to compare the degree of Dox protein binding as compared with unprocessed samples, using the same microbiological analysis. The percentage of protein-bound fraction (B) was calculated according to the following equation: B = (initial plasma concentration - ultrafiltrate concentration)/initial plasma concentration × 100. The CV for this method were <4.2%.

## Results

http://Figure 1 shows the mean ± 1 SD of the serum concentrations of Dox vs time for the three drug preparations (Dox-β, Dox-pol and Dox-a). Table 1 summarizes the pharmacokinetic variables obtained and statistical differences are highlighted.

Maximum serum concentration (Cmax ) was highest in the Dox-pol group (1.3 ± 0.7 μg/mL) at a Tmax of 5.9 ± 1.7 h. Elimination half-life (T½β) in the Dox-pol group was 4.9 ± 1.0 h, a similar value was obtained for Dox-β (4.2 ± 0.9 h), while T½β determined after the administration of Dox-a was 2.8 ± 0.9 h. Dox-β had considerably lower plasma concentrations throughout the established sampling period as compared to the other two groups. The AUC for plasma concentrations was higher for Dox-pol (17 ± 2.2 μg h/ml), intermediate for Dox-a (3.1 ± 0.2 μg h/ml) and lowest for Dox- β (1.5 ± 0.1) (P < 0.01). Plasma protein binding did not differ among groups and was consistently 80.3 ± 1.5%.

Relative bioavailability of the two long-acting preparations as compared to Dox-a was 548% for Dox-pol and 48% for Dox-β (P < 0.01).

## Discussion

Agreement between the quantitative/qualitative microbiological agar diffusion technique and the high performance liquid chromatography method used in this trial to determine serum concentrations of Dox, can be regarded as sufficiently reliable to assume that concentrations obtained through HPLC are biologically active. That is, because the microbiological agar diffusion test determines the active fraction(s) of the drug, it offers clinically meaningful data, and in this case such assumption has been validated through a purely chemical method. This allows straightforward speculations on the relationships between serum concentrations and dosing intervals for specific pathogens.

Serum concentrations obtained after the administration of Dox-β were noticeably low (Cmax = 0.2 ± 0.0 mg/mL; AUC = 1.5 ± 0.1 μg · h/mL). These values are not within the range that would be effective for many equine pathogens [1,22], and are only considered marginally for further analysis in this section.

The pharmacokinetics of the aqueous solution of Dox-h administered orally has been determined in adult horses [1,6,11,12] at a dose range from 3 to 20 mg/kg. Davis et al. [6] obtained a Cmax of 0.9 ± 0.2 μg/mL with a Tmax of 1.6 ± 1.3 h, administering a single dose of 20 mg/kg PO. By comparison Cmax obtained with Dox-a in this study, at a single 10 mg/kg dose was 0.3 ± 0.1 mg/mL with a Tmax of 2.2 ± 0.4 h. Differences between these two studies can be safely related to the dose and biological variability, but it relates mainly to the fact that the former study used fasted horses. In contrast, Cmax and Tmax values for Dox-pol were 1.3 ± 0.6 μg/mL and 5.9 ± 1.6 h, respectively, also at a dose of 10 mg/kg. By comparison higher Cmax at a lower or the same dose may be the result of a concentration build-up due to a zero order absorption kinetics caused by the polymers in the preparation along a greater absorption surface area of the GI tract. This would also explain the longer Tmax observed. The AUC obtained administering 20 mg/kg as reported by Davis et al. [6] was 13.3 ± 2.7 μg.h/mL, while the AUC obtained with the Dox-pol formulation at half their dose was 17.0 ± 2.2 μg.h/mL. This finding is not unusual for LA preparations that exhibit flip-flop kinetics [23] and may also explain the relative bioavailability which reaches an unusual 548%. In turn, to demonstrate flip-flop pharmacokinetics, the overall appearance of the serum concentration vs. time profile of the drug must be accounted for. Occasionally, as in this case, the slower rate of absorption as compared to the rate of elimination is not a straightforward conclusion to be drawn. If a much longer apparent elimination half-life following extravascular dosing is observed compared with the IV route, it suggests that flip-flop pharmacokinetics is occurring [24]. However this is not possible with Dox considering that IV administration of this drug in horses is not recommended [9,10]. Thus, applying the following equation and based on information taken from published work [25,26], a flip-flop condition may be demonstrated with the following equation:

$$\text{Rate}\;\mathrm{of}\;\text{Absorption}=\mathrm{Vz}\;(\mathrm{KC}+\left(\mathrm{\Delta C}/\mathrm{\Delta t}\right)$$

Where Vz is the terminal exponential volume of distribution, K is the terminal disposition rate constant once drug absorption is complete, C is the plasma concentration at time t and ΔC is the change in plasma concentration over the time interval Δt. For Dox-pol plasma concentration-time data at 4 and 12 h , ΔC/Δt = 0.0176 μg/mL/h. At the midpoint of this time period (8 h), (K)(C) = 0.1339 μg/mL/hr. Since KC > ΔC/Δt, rate of absorption ≈ rate of elimination, a “flip-flop” condition exists and the Dox-pol formulation here described can be regarded as a true long-acting one.

As far as Dox-pol is concerned, poloxamer 407, a polyethylene oxide-polypropylene oxide- polyethylene oxide triblock co-polymer, was used as delivery vehicle-matrix. It has been shown that it enhances solubility and permeability, often resulting in improved oral bioavailability, as reported by Kahn et al. [27] using atorvastatin, carbamazepine and other poorly soluble drugs. Considering the permeability and solubility rates of doxycycline, there is controversial information regarding the classification of this drug in the Biopharmaceutics Classification System (BCS) for humans [28]. Initially Amidon et al., [29] classify this drug in Class IV, that is, poorly soluble and poorly permeable. More recently, however, Chavda et al. [30] include Dox in Class I; that is, Dox is highly soluble and highly permeable, which shows no coherence when analyzing its reported absolute bioavailability in horses i.e., from 2.8% to 17% [12,31]. In this study, absolute bioavailability of Dox for the two long-action formulations (Dox-pol and Dox-β) was not determined because the IV kinetics of the drug is needed, and risk of cardiovascular toxicity was avoided [10]. The F_rel_ for Dox-pol and Dox-β was 548% and 48%, respectively, as compared to Dox-a, in non-fasted horses. Even though decreased oral absorption of Dox has been demonstrated in fed horses [31,32], it is here theorized that other factors could have enhanced bioavailability of Dox administered in the Dox-pol formulation: mucoadhesiveness achieved by poloxamer, carbopol and xanthan gum in the formulation [33-35], perhaps enterohepatic circulation considering that, with few exceptions, this phenomenon is recognized as a common physiological peculiarity of tetracyclines [36], and a greater amount of fluid participating to produce better Dox dissolution in the horses’ entire gastrointestinal lumen. This may allow absorption along other surfaces of the GI tract [31]. Additionally, total transit time from mouth to colon in adult horses is of approximately 40 h [37], a fact that complies well with the former reasoning. However, further studies are warranted to define these phenomena.

The benefits of the controlled delivery of drugs include: the maintenance of serum drug concentration at an optimal therapeutic level for a more prolonged time-interval, reduction in handling and consequently, a possible improvement in drug-administration compliance [38]. In this context, the Dox-pol preparation here described was capable of providing with a single oral administration, useful serum concentrations of this antibacterial drug for 24 h, but not longer. Although it has been stated that high concentrations of Dox *in vitro*, equivalent to 8 to 16 times the value of an average MIC could turn this time-dependent antibacterial drug into a concentration-dependent antibacterial drug [39], its cardiac toxicity refrains its use at higher doses. Hence, seeking large Cmax values is an unsafe approach [40,41]. Dox should be considered a time-dependent antibacterial drug in horses. In that context a better PK/PD ratio can be achieved when serum concentrations of the drug are barely above or at the MIC level of the involved pathogen for as long as possible within the dosing interval [36,42]. Values of MIC that can be adopted in this trial can be set from 0.25 to 1.0 μg/mL [6,22] Hence, the length of time in which minimum therapeutic concentrations can be achieved with Dox-pol varies from 12 to 24 h. Additionally, in humans, a PK/PD index accepted as predictor of therapeutic efficacy for tetracyclines as a group is the ratio of AUC0-24 (AUC0-24)/MIC [43]. If a MIC of 0.25 μg/mL is considered, the AUC_DOX-pol_/MIC ratio is 68.02 and the AUC_DOX-a_/MIC ratio is only 12.4. Considering the above, it is safe to regard Dox-pol as a drug preparation that possesses better PK/PD ratios to control bacterial diseases in horse as compared to Dox-a.

## Conclusions

Dox-pol is an oral paste formulation that optimizes the use of doxycycline in horses in terms of PK/PD ratio congruency, and it is likely that it may also improve prescription compliance, due to its ease of administration. This may contribute to diminish the emergence of bacterial resistance. Nevertheless and although no adverse gastrointestinal reactions (diarrhea, colic, abdominal discomfort) were observed in any of the horses used in this trial, multiple dose, tissue distribution and toxicological studies are needed before clinical trials are set, to assess if this preparation can be regarded as potentially useful in this species.

## Competing interests

The author(s) declare that they have no competing interests.

## Authors’ contributions

HS and LG participated in the study design, planning and coordination, performed the statistical analysis and helped to draft the manuscript. HZ prepared and administered the formulations, carried out the blood sampling and was in charge of analytical procedures; she also helped to draft the manuscript. MJB participated in the study design and coordination as well as supervision of the chemical soundness of formulations. All authors read and approved the final manuscript.
